# Why These Errors?

**Published:** 1887-11

**Authors:** H. Hitchcock


### WHY THESE ERRORS?

Will some one please explain why these discrepancies should occur in such works as:

| Hering's Condensed Materia Medica? | Hering's Guiding Symptoms? |
|---|---|
| **Ammonium Carbonicum.** | |
| Hypochondria—Pressure or sore feeling in right hypochondrium.<br>Stitches in left hypochondrium. | Hypochondria—Pressure or sore feeling in hypochondrium.<br>Stiches in hypochondrium. |
| Stool—Discharge of blood before and after stool.<br>Discharge of prostatic fluid after stool. | Stool and Rectum.* |
| Urine—Involuntary urination during sleep, toward morning. | Urinary Organs—1 Involuntary urination during sleep. |
| Sexual Organs, Female—Menses ***; —very slightly colored. | Female Sexual Organs—Menses ***. |

---

* Symptomen Codex gives: *After the evacuation:* ***; discharge of a milky prostatic fluid; **discharge of blood during and after evacuation.*

| | |
|---|---|
| Cough—At night, about midnight. Violent about three or four A. M. | Cough—Dry cough at night with tickling, etc.<br>2 Cough at night; every morning at three o'clock, etc. |
| Outer Chest—Right mamma painful to touch. | Outer Chest. |
| Skin—Upper half of body as red as scarlet. | Skin. |

H. HITCHCOCK, M. D.

[Notwithstanding these slight discrepancies, *The Guiding Symptoms of the Materia Medica* is incomparably the best work yet published, *The North American Journal of Homœopathy* to the contrary notwithstanding. This strictly (?) Hahnemannian organ thinks that *The Guiding Symptoms* "lead to empiricism and allopathy." No doubt! It is probably a great deal more conducive to the practice of pure Homœopathy to give Quinine in massive doses in intermittents, Morphia for pain, disinfectants for ulcers, etc., as is done by nine out of ten of the men calling themselves homœopathists. Nevertheless, we shall continue to recommend *The Guiding Symptoms* to our readers as worthy of their highest confidence.—EDS.]

